# Feed containing heat‐killed lactic acid bacteria as a single species or multispecies combination improves feeding, defense, and stress in coho salmon

**DOI:** 10.14814/phy2.70324

**Published:** 2025-04-13

**Authors:** Kiyomi Takase, Yoshio Hirasawa, Izuru Kakuta

**Affiliations:** ^1^ Tohoku Seikatsu Bunka University Junior College Sendai Miyagi Japan; ^2^ TSI Company Limited Hongo, Bunkyo‐Ku Tokyo Japan; ^3^ Faculty of Science and Engineering, Ishinomaki Senshu University Ishinomaki Miyagi Japan

**Keywords:** coho salmon, heat‐killed bacteria, *Lactobacillus delbrueckii*, *Lactococcus lactis*, *Leuconostoc mesenteroides*, multispecies combination

## Abstract

There are few reports examining the effects of simultaneous administration of multiple killed lactic acid bacteria with different physiological properties to fish. In this study, we fed three heat‐sterilized lactic acid bacteria, *Lactobacillus delbrueckii* (Lac‐b‐d), *Leuconostoc mesenteroides* (Leu‐m), and *Lactococcus lactis* (Lac‐c‐l), either singly or in combination (Combination), to juvenile coho salmon *Oncorhynchus kisutch* for 4 weeks and examined the changes in the physiological responses of the fish. At a daily bacterial intake of 0.1 mg/kg BW, granulocyte increased in the Leu‐m, Lac‐c‐l, and Combination groups; lymphocyte increased in the Lac‐c‐l group; and granulocyte phagocytic activity was higher in the Lac‐b‐d and Combination groups. In a 4‐week feeding experiment in which the intake of the multispecies Combination was varied, a feeding attraction effect was found in the 0.05 and 0.1 mg/kg BW/day groups, red blood cell and lymphocyte counts, granulocyte phagocytic activity, and potential killing activity were significantly higher in the combination group than in the control group. The survival rate in hypoxic conditions was also remarkably higher in the 0.05 and 0.1 mg/kg BW/day combination groups. These results suggest that the administration of multiple heat‐killed lactic acid bacteria is effective in improving the physiological responses of fish.

## 1 INTRODUCTION

Aquaculture, including fish farming, is often carried out in fixed cages in narrow bodies of water. In addition, any outbreak of bacterial, viral, and parasitic diseases in the surrounding waters or within the cages can result in a significant increase in the number of infected fish in a short period of time, due to the high density of fish in the cages.

In recent years, in addition to various aquatic animal medicines (antibiotics, antibacterial agents, anthelmintics) mainly for the purpose of treatment, highly effective vaccines for the purpose of infection prevention have been developed. However, there are few approved commercially available vaccines, and the vaccines that can be used are limited in terms of the breeding conditions of the target fish species and their usage, and depending on how they are used, they are not necessarily highly effective (Rathor & Swain, 2024; Wakabayashi, 2002). For these reasons, even now when the effectiveness of vaccines is widely recognized, they have not yet been able to completely prevent the onset of diseases or cure the disease. Attempts are being made to eliminate or reduce not only disease but also various physiological disorders and stress loads associated with environmental changes by daily administering various supplements (nutritional supplements), useful microorganisms, bioactive peptides, polyphenols, DHA (Docosahexaenoic Acid) and EPA (Eicosapentaenoic Acid), and herbs. However, the effects of many of these substances are not fully clear, and some contain harmful ingredients. Furthermore, the possibility that substances administered in the hope of being effective may remain in the fish meat or may cause adverse effects on the living organisms or the environment due to the emergence of drug‐resistant bacteria cannot be ignored.

Lactic acid bacteria have been widely used in research to improve the bio‐defense mechanisms of humans and animals by providing them feed or water containing live bacteria (Gildberg & Mikkelsen, 1998; Takada et al., 2006). The classification of lactic acid bacteria includes many types of microorganisms, but among the homolactic acid bacteria *Lactococcus*, *Enterococcus*, *Streptococcus*, and *Pediococcus*, as well as some *Lactobacillus*, and the heterolactic acid bacteria *Leuconostoc*, some of which have been administered to fish and have shown positive results, such as antibacterial and bacteriostatic activity, increased defense mechanisms, improved intestinal function, and growth promotion. For example, the administration of live *Lactobacillus* is known to have immunostimulatory or disease‐resistance effects in fish (Byun et al., 1997; Gatesoupe, 1994; Gildberg & Mikkelsen, 1998; Mohammadian et al., 2024; Takada et al., 2006). There are also reports that the administration of *Lactobacillus delbrueck*ii was effective in promoting fish growth (Mohammadian et al., 2024). Administration of *Lactococcus lactis* leads to the activation of the biological defense function of fish (Phinyo et al., 2024; Wu et al., 2023), and it has been reported that a strain of this bacterium has the effect of promoting fish growth (Xia et al., 2018). It has also been suggested that the administration of *Leuconostoc mesenteroides* enhances the antimicrobial activity of fish (Xia et al., 2024). Furthermore, there have been reports that the administration of a mixture of live multiple bacteria improves the health of livestock (Giang et al., 2012; Okabe et al., 2003).

However, to improve the health of fish using live lactic acid bacteria, it is necessary to feed the fish with the bacteria prepared according to the timing of feeding, which is very difficult in terms of cost and labor. It has also been reported that it is difficult to adhere live lactic acid bacteria to the intestinal epithelial cells (Ohashi, 2006). In other words, the use of killed bacteria leads to improved convenience, such as a long shelf life and easy storage and transportation. It has also been reported that the components of lactic acid bacteria themselves have the effect of stimulating intestinal cells and changing the balance of intestinal bacteria (Kaji, 2012) and activating macrophages (Kawamura et al., 2000). In recent years, therefore, there have been increasing attempts to use lactic acid bacteria not only as live bacteria but also as killed bacteria (Bernardeau & Vernoux, 2024; Ringø et al., 2020; Tran et al., 2020).

There are several cases where the expected effects were not achieved by ingestion of live bacteria, but using killed bacteria has been effective. For example, *Enterococcus faecalis* FK‐23 strain isolated from the feces of healthy individuals has not been shown to have any immunostimulatory effect on mammals as a live bacterium, but the heat‐killed bacteria have been reported to have beneficial effects, such as activating neutrophils and macrophages in vitro (Ohasi et al., 1993), promoting the increase of white blood cells, and suppressing infectious diseases (Hasegawa et al., 1994). Investigation on the effect of administering live and killed *Lactobacillus plantarum* Zhang‐LL on alleviating the symptoms of dextrin sulfate sodium‐induced colonic ulceration in rats showed that administration of killed bacteria had a higher protective effect in many respects than administration of live bacteria (Jin et al., 2020). In fish, it has been reported that the administration of heat‐killed *E. faecalis* FK‐23 improves the vaccination effect in flounder (Kotani et al., 2008) and enhances the innate immune activity and antioxidant activity in rainbow trout (Kakuta & Takase, 2024). It has also been found that oral administration of a large amount of *E. faecalis* FK‐23 (600 mg/kg body weight/day) appears to improve lipid metabolism through regulating the intestinal flora of rainbow trout (Kakuta & Takase, 2024).

The reasons for choosing juvenile coho salmon, *Oncorhynchus kisutch*, as the subject of this study are as follows: Freshwater‐cultured coho salmon are transported to marine farming areas between October and December, where they are cultured on the sea surface until early summer of the following year. However, in recent years, Miyagi Prefecture has been experiencing high water temperatures in freshwater areas, as well as the spread of infectious diseases, and a shortage in the supply of juvenile fish has become an issue. In other words, there is a need for methods and administration materials to improve the biodefense activity and stress resistance of juvenile fish. In this study, therefore, we used coho salmon to investigate the effects of feed including one of each and a multispecies combination of heat‐killed lactic acid bacteria, *Lactobacillus delbrueckii* NBRC116386, *Lactococcus lactis* NBRC116387, and *Leuconostoc mesenteroides* NBRC116388, which have been reported to have various functions such as enhancing biodefense activity, regulating the intestines, and promoting growth, on the physiological responses such as feeding behavior (feeding attraction effect), bio‐defense activity and stress resistance.

## 2 MATERIALS AND METHODS

### 2.1 Ethics statement

This study was conducted at Department of Biological Science, Ishinomaki Senshu University, Japan. All experimental protocols and methods were conducted in accordance with “Raising laboratory animals Standards for storage and pain reduction” by the Ministry of the Environment of Japan.

### 2.2 Strain and preparation of bacteria and preparation of heat‐killed lactic acid bacteria

*Lactobacillus delbrueckii* NBRC116386 (from Sargassum *Sargassum fulvellum*), *Leuconostoc mesenteroides* NBRC116387 (from Sargassum *Sargassum fulvellum*), and *Lactococcus lactis* NBRC116388 (from sea sand) were inoculated into MRS liquid medium (MRS broth, BD Difco) and cultured at 30°C for 48 h, and then inoculated into soy milk medium (solid concentration 9%) (SOYMILK UNSWEETENED domestically‐produced soybeans, MARUSAN‐AI CO., LTD.) to a concentration of 10^6^ cells/mL and cultured at 30°C for 48 hours.

Heat‐killed bacteria were prepared from the above three types of lactic acid bacteria obtained by culture as follows. That is, the bacteria were cultured in MRS liquid medium at 30°C for 48 h and then cultured on soy milk medium at 30°C for 48 h, after which the bacteria were collected. And then, dextrin (Starch Decomposition Product, Matsutani Chemical Industry Co., Ltd.) was added to adjust the viscosity and particle size so that the Brix value was about 20%, and the bacteria were sterilized in a water bath at 80°C for 75 min. After the heat treatment, the bacteria were immediately cooled and dried at around 80°C using an L‐8 type spray dryer (OHKAWAHARA KAKOHKI Co., Ltd.) to obtain a powder containing dead bacteria. The number of bacteria in the prepared heat‐killed lactic acid bacteria powder was 2.0 × 10^12^ cells/g for *Lactobacillus delbrueckii* NBRC (Biological Resource Center, NITE [NBRC] Culture Catalogue) 116,386, 2.0 × 10^12^ cells/g for *Leuconostoc mesenteroides* NBRC116387, and 3.0 × 10^12^ cells/g for *Lactococcus lactis* NBRC116388. These were mixed in a ratio of 2:2:3 to prepare a heat‐killed bacteria additive.

### 2.3 Experimental fish and rearing conditions

Juvenile coho salmon *Oncorhynchus kisutch* (average body weight about 45 g) were used as test fish. Depending on the test, two to five 100‐liter rearing containers with mesh on four sides were placed afloat in a 3.5‐ton constant temperature circulating filtration elliptical tank, and 15 fish were placed in each container. The fish were given control feed (trout feed mixture [Feed One Trout EP Puff Clean, FEED ONE Co., Ltd.] crushed and reshaped into pellets: crude protein 45.0% or more, crude fat 9.0% or more, crude fiber 4.0% or less, crude ash 12.0% or less) at a daily rate of 2.0% of the fish's body weight. After 2 weeks of preliminary rearing, the feeding experiment began. The feeding conditions during the feeding trial were as follows: Coho salmon were given 2.0% of their body weight per day (the control group was given trout feed [Feed One's Trout EP Puff Clean] crushed and reshaped into pellets [the same as during preliminary rearing]), and the four test groups were fed trout feed with three types of killed lactic acid bacteria added in a specified amount, either one of each or a multispecies combination, and remolded into pellets, 5 days a week, at around 10 am and 4 pm, for a 4‐week feeding test.

First, to understand the effect of administration of dextrin, which was used as a viscosity and granularity adjusting material in the preparation of the powder containing heat‐killed lactic acid bacteria cells, on the feeding behavior, survival rate and biodefense‐related indicators of the fish, a diet containing 0.50% of the material was prepared and administered (the daily dose of dextrin for a 1 kg fish was 100 mg). The duration of this experiment was 4 weeks, the rearing water temperature was 16°C, the water oxygen concentration was approximately 8.0 mg/L, and the daily feeding amount was 2.0% of body weight.

In Experiment I, either one of each or a multispecies combination administration test of the three types of heat‐killed lactic acid bacteria, the juvenile coho salmon were divided into five groups: a control group, groups administered single killed cell powders of *Lactobacillus delbrueckii* NBRC116386 (Lac‐b‐d group), *Leuconostoc mesenteroides* NBRC116387 (Leu‐m group) *and Lactococcus lactis* NBRC116388 (Lac‐c‐l group), and a group administered the mixture of killed lactic acid bacteria powders (Combination or M group). The amount of powder containing heat‐killed lactic acid bacteria cells added to the feed was 0.5% (the amount of heat‐killed lactic acid bacteria cells administered to a 1 kg coho salmon per day was 0.1 mg for each group, the number of cells in the single administration groups was 2.0 × 10^8^, 2.0 × 10^8^, and 3.0 × 10^8^, and the number of cells in the multispecies combination administration groups was 0.67 × 10^8^, 0.67 × 10^8^, and 1.0 × 10^8^ of each of the aforementioned bacteria, corresponding to a total of 2.3 × 10^8^).

In the next test (Experiment II) to evaluate the optimal dosage of the mixture of three types of heat‐killed lactic acid bacteria, in addition to the control group, the feed was supplemented with a combination of the three types of heat‐killed lactic acid bacteria powders at 0.125, 0.25, 0.5, and 1.0% (the daily dosage of the heat‐killed lactic acid bacteria per 1 kg of coho salmon was 0.025, 0.05, 0.1, and 0.2 mg, respectively). The water temperature during the preliminary and normal rearing periods was 16°C, and the dissolved oxygen concentration in the water was 8.0–8.5 mg/L.

### 2.4 Investigation of feeding behavior

Feeding behavior was investigated from the start of rearing to the day before the end of rearing. As an index, the time it took to finish the daily amount of feed was measured, and positive integers were assigned in ascending order from the group that finished eating the most quickly (feeding attraction effect; It's term means feeding behavior became more active and the time it took to finish eating a certain amount of food became shorter). Throughout the test period, the order of feeding completion for each feeding day was calculated, and feeding attraction effect was evaluated from the order of feeding completion (group that finished eating the fastest) throughout the feeding period. In Experiment I, feeding attraction behavior was examined for 20 days selected at random during the 28‐day rearing period, excluding Saturdays and Sundays, and in Experiment II, feeding attraction behavior was examined for 38 days from the start to the end of feeding (feeding was 5 days a week, excluding Saturdays and Sundays, so the total number of feeding days was 28 days).

### 2.5 Measurement of physiological and biodefense indices

After the feeding experiment was completed, five fish were randomly taken from each group, blood was collected from the tail vessel of coho salmon using a heparinized syringe, and the biodefense activity and the health indices in the blood were measured. That is, the number of red blood cells (RBC) was determined by the blood cell calculator method after dilution of whole blood with 0.75% NaCl. The blood was smeared on a glass slide, stained with May‐Grünwald's stain solution (Code No. 131–12,811, FUJIFILM Wako Pure Chemical Industries, Ltd.), and examined to measure the ratio of granulocytes and lymphocytes per RBC, and then the actual number of each blood cell was calculated using the number of RBC measured separately. Saline suspended with 0.6 mg/mL of zymosan A (from *Saccharomyces cerevisiae*, CAS No. 58856–93‐2, Sigma‐Aldrich Japan) was mixed with whole blood in equal volumes, and the mixture was allowed to react for 30 min at 25°C with inversion and mixing every 5 min. The ratio of zymosan‐incorporated granulocytes per 100 granulocytes was determined.

In addition, the potential killing (PK) activity of the blood and the lysozyme activity of the mucus on the surface of the body were measured. The former was determined according to the usual method (Miyazaki, 1998). That is, it was determined by calculating the difference in absorbance between the zymosan A suspension and the NBT solution. The latter (only performed in Experiment II) was examined by scraping the mucus on the surface of the fish with a microspatula and examining the bacteriolytic status of *Micrococcus luteus* (*Micrococcus lysodeikticus* ATCC No. 4698, Sigma‐Aldrich Co., Ltd.) at pH 6.8 in the usual manner (the rate of decrease in absorbance at 620 nm per hour converted to 1 g of protein in the mucus) (Kakuta & Takase, 2024).

### 2.6 Hypoxic load test

Under normal dissolved oxygen conditions at a water temperature of 16°C, the groups (each group housed 20 individuals) that had been subjected to the same four‐week feeding experiment as described above were divided into two groups of 10 individuals each, and one group was kept in the same environment as before, while the other group was kept under hypoxic conditions with a dissolved oxygen concentration of 4.2 ± 0.2 mg/L for 4 days each (hypoxic load test). Hypoxic tolerance was evaluated based on the survival status of each group three and 4 days after the start of the test. No food was given during this test.

### 2.7 Statistics

In principle, the tables and figures in the text are shown as mean values ± standard deviations unless otherwise stated. The sample size for comparing the mean values between the two groups was calculated using the free software EZR under the following conditions: Type I error: 0.05, power: 0.08, sample size ratio: 1:1. Tukey's multiple range test was used for multiple comparisons based on one‐way ANOVA, with *p* < 0.05 as the limit of significance. Regarding the feeding attraction effect, the presence or absence of significant differences was determined using the Steel‐Dwass multiple comparison test (*α* < 0.05).

## 3 RESULTS

### 3.1 Understanding the effect of administration of dextrin for viscosity‐ and particle‐size‐adjusting on the growth and physiological indices of coho salmon

Table 1 shows the effect of administration of dextrin on the feeding behavior, growth, and physiological indices of red and white blood cell counts and some kinds of bio‐defense activity indices of coho salmon. No difference was observed between the control and the dextrin‐administered groups in any of the items investigated.

**TABLE 1:** Effects of feed including dextrin added to adjust the viscosity and particle size of bacterial powder on the body weight, body length, and the condition factor, feeding activity, and some physiological indices of coho salmon, *Oncorhynchus kisutch*.

	Control	Dextrin
BW (g)	62.8 ± 10.0	68.1 ± 14.4
BL (mm)	167 ± 10	175 ± 11
CF	13.4 ± 0.8	12.6 ± 0.4
Feeding activity	1.15 ± 0.37	1.25 ± 0.44
RBC (×10^5^/μL)	14.9 ± 4.6	13.3 ± 4.5
Lymphocyte (×10^3^/μL)	2.05 ± 0.72	2.05 ± 1.19
Granulocyte (×10^3^/μL)	1.29 ± 0.32	1.31 ± 0.51
Phagocytic activity (%)	16.9 ± 2.0	16.9 ± 0.51
Potential killing activity (O.D. 620 nm)	0.024 ± 0.008	0.027 ± 0.015

*Note*: The daily dose of dextrin #2 powder (Starch Decomposition Product, Matsutani Chemical Industry Co., Ltd.) to a coho salmon weighing 1 kg is 100 mg. Data are given as mean ± SD, *n* = 7 (Control group) or 5 (Dextrin group).

Abbreviation: CF, Condition factor = BW × 100,000/(BL)^3^.

### 3.2 Effects of feeding a fixed amount of heat‐killed lactic acid bacteria either one of each or a multispecies combination on the growth and physiological indices of coho salmon (Experiment I)

The survival rate of the control group and the four test groups administered three kinds of heat‐killed lactic acid bacteria, either one of each or the combination, at the daily intake of 0.1 mg/kg body weight during the preliminary rearing and rearing tests was 100%, and no abnormalities were observed in behavior and external morphology, and the feeding conditions were good in all groups.

Table 2 shows the results of an investigation into the feeding attraction effect of heat‐killed lactic acid bacteria when fed alone or in combination for 20 days randomly selected during the 28‐day rearing period (Experiment I). The lower the number shows the higher the feeding attraction effect. The groups administered heat‐killed *Lactobacillus delbrueckii*, heat‐killed *Leuconostoc mesenteroides*, heat‐killed *Lactococcus lactis*, and the multispecies combined bacteria had many smaller numbers than the control group, but no statistically significant differences were observed between the groups in this item.

**TABLE 2:** Effect of feed including heat‐killed lactic acid bacteria, either singly or in combination, on the feeding attraction effect of coho salmon *Oncorhynchus kissachi* (upper; the number of times each rank of the 20 surveys conducted, lower; statistical dataα).

Experimental group	Feeding completion rank				
	1	2	3	4	5
Control	6	1	6	3	4
*L. delbrueckii* (Lac‐b‐d)	11	2	2	3	2
*L. mesenteroides* (Leu‐m)	10	3	4	2	1
*L. lactis* (Lac‐c‐l)	14	1	2	2	1
Multispecies combination	13	1	4	1	1

	Control	L‐b‐d	Leu‐m	Lac‐c‐l
L‐b‐d	0.41990	ー	ー	ー
Leu‐m	0.29243	0.99936	ー	ー
Lac‐c‐l	0.06866	0.88718	0.95764	ー
Combination	0.09055	0.92757	0.97812	0.99996

*Note*: Lac‐b‐d: Lactobacillus delbrueckii NBRC116386. Leu‐m: Leuconostoc mesenteroides NBRC116387. Lac‐c‐l: Lactococcus lactis NBRC116388. Multispecies Combination: Lac‐b‐d: Leu‐m: Lac‐c‐l = 2: 2: 3 (Mixture). The daily dose of either one of each or a multispecies combination of the three kinds of heat‐killed lactic acid bacteria to a coho salmon weighing 1 kg is 0.1 mg. The presence or absence of significant differences was determined using the Steel‐Dwass multiple comparison test (significance level *α* < 0.05).

Table 3 shows the effect of the administration of either one of each or a multispecies combination of heat‐killed lactic acid bacteria on the growth and red blood cell count of coho salmon. There was no statistically significant difference in body weight, body length, and condition factor of fish between the control and the test groups. The number of red blood cells in the group administered heat‐killed *Lactococcus lactis* was slightly higher than that in the control. Due to the large individual differences in each group, however, no statistically significant differences were observed between the control and the test groups.

**TABLE 3:** Effects of feed including heat‐killed various lactic acid bacteria on the body weight, body length, and the condition factor in coho salmon, *Oncorhynchus kisutch* (Experiment I).

	Control	Lac‐b‐d	Leu‐m	Lac‐c‐l	Combination
BW (g)	59.0 ± 14.8	58.9 ± 5.1	68.1 ± 11.5	53.9 ± 6.8	50.8 ± 3.8
BL (mm)	164 ± 13	167 ± 4	174 ± 9	164 ± 10	161 ± 5
CF	13.3 ± 0.7	12.6 ± 0.4	12.9 ± 1.6	12.3 ± 1.1	12.3 ± 1.4
RBC (×10^5^/μL)	13.5 ± 5.9	13.7 ± 5.1	11.8 ± 2.6	17.0 ± 3.5	13.3 ± 4.5

*Note*: Lac‐b‐d: *Lactobacillus delbrueckii* NBRC116386. Leu‐m: *Leuconostoc mesenteroides* NBRC116387. Lac‐c‐l: *Lactococcus lactis* NBRC116388. Combination: Lac‐b‐d: Leu‐m: Lac‐c‐l = 2: 2: 3 (Mixture). The daily dose of either one of each or a multispecies combination of the three kinds of heat‐killed lactic acid bacteria to a coho salmon weighing 1 kg is 0.1 mg. Data are given as mean ± SD, *n* = 5.

Abbreviation: CF, Condition factor = BW × 100,000/(BL)^3^.

**FIGURE 1:**
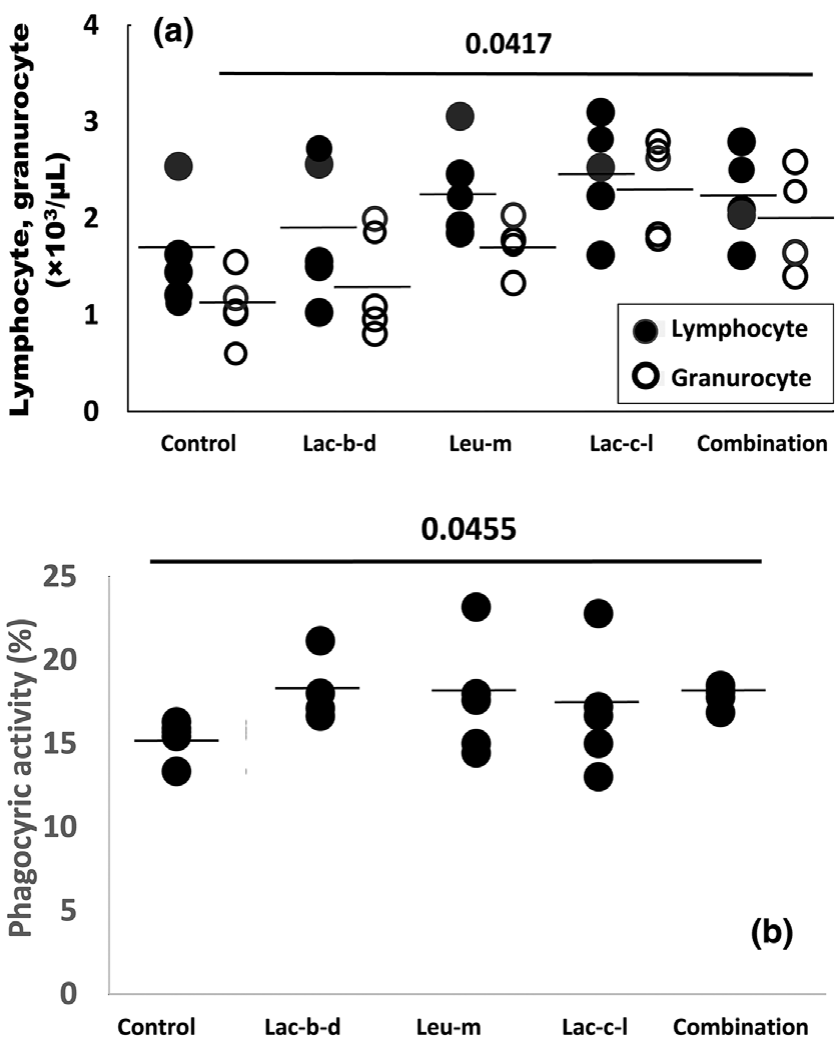
Effect of feed including heat‐killed various lactic acid bacteria on the number of red blood cells (a), granulocytes and lymphocytes (b) in coho salmon, *Oncorhynchus kisutch*. Lac‐b‐d: *Lactobacillus delbrueckii* NBRC116386. Leu‐m: *Leuconostoc mesenteroides* NBRC116387. Lac‐c‐l: *Lactococcus lactis* NBRC116388. Combination: Lac‐b‐d: Leu‐m: Lac‐c‐l = 2: 2: 3 (Mixture). The daily dose of each heat‐killed lactic acid bacterial powder to a coho salmon weighing 1 kg is 100 mg (the daily dose of the heat‐killed lactic acid bacteria is 0.1 mg). Data are given as mean ± SD, *n* = 5. *p*‐ value are by one‐way ANOVA with Tukey's post‐test for multiple comparisons.

Figure 1 shows the effect of feeding either one of each or a multispecies combination of heat‐killed lactic acid bacteria on the lymphocyte and granulocyte counts (a), and on the phagocytic activity of granulocytes in coho salmon. Lymphocyte counts tended to increase in the groups fed heat‐killed *Leuconostoc mesenteroides* and *Lactococcus lactis*, and in the multispecies combination. Granulocyte counts increased significantly in the group fed heat‐killed *Lactococcus lactis* (*p* = 0.0417). The phagocytic activity of the multispecies combination group was significantly higher than the control group (*p* = 0.0455). Although the average values of the groups administered heat‐killed *Leuconostoc mesenteroides* or *Lactococcus lactis* were 1.11 to 1.15 times higher than the control, no significant difference was observed between the control and the test groups.

The potential killing (PK) activity values of the groups administered either one of each or a multispecies combination of heat‐killed lactic acid bacteria were slightly higher than those of the control group, but due to the large individual differences in each group, no statistically significant difference was observed between the control and the teat groups (the daily dose of the heat‐killed lactic acid bacteria is 0.1 mg/kg body weight). Data (*n* = 5) were of each group as follows: Control; 0.025 ± 0.006, Lac‐b‐d; 0.036 ± 0.030, Lue‐m; 0.036 ± 0.019, Lac‐c‐1; 0.043 ± 0.037, Combination; 0.031 ± 0.016.

### 3.3 Evaluation of the optimal dosage of a multispecies combination of heat‐killed lactic acid bacteria (Experiment II)

The survival rate of the control and the four test groups in which the daily intake of the combination of heat‐killed lactic acid bacteria was set at 0.025, 0.05, 0.1, and 0.2 mg per kg of fish body weight during the rearing tests was 100%, and no abnormalities were observed in swimming performance, feeding behavior, and external morphology.

Table 4 shows the effect of the administration of different amounts of the heat‐killed lactic acid bacteria on the growth of coho salmon. There was no statistically significant difference in body weight, body length, and condition factor between the control and the four test groups administered different amounts of the bacteria.

**TABLE 4:** Effects of feed including the heat‐killed lactic acid bacteria mixture with a ratio of *Lactobacillus delbrueckii* NBRC116386, *Leuconostoc mesenteroides* NBRC116387, and *Lactococcus lactis* NBRC116388 of 2:2:3 on the body weight, body length, and the condition factor in coho salmon, *Oncorhynchus kisutch* (Experiment II).

	Control	25 M (0.025)	50 M (0.05)	100 M (0.1)	200 M (0.2)
BW (g)	63.4 ± 8.6	60.9 ± 6.3	73.1 ± 10.3	62.8 ± 13.9	63.6 ± 12.8
BL (mm)	162 ± 8	160 ± 9	167 ± 6	165 ± 10	160 ± 7
CF	14.8 ± 0.8	15.0 ± 2.5	15.5 ± 0.6	13.8 ± 1.1	15.3 ± 1.5

*Note*: The number before M indicates the amount of heat‐killed lactic acid bacterial powder to be administered to 1 kg of fish per day. The number in parentheses indicates the amount of heat‐killed lactic acid bacteria to be administered to 1 kg of fish per day. Data are given as mean ± SD, *n* = 5.

Abbreviation: CF, Condition factor = BW × 100,000/(BL)^3^.

Table 5 shows the effect of administration of different amounts of the heat‐killed lactic acid bacteria combination on the feeding behavior of coho salmon. In the groups of coho salmon that received 0.05 and 0.1 mg of the combination of heat‐killed *Lactobacillus delbrueckii*, *Leuconostoc mesenteroides*, and *Lactococcus lactis* per kg of body weight per day, the values were significantly lower than those of the control. This change was observed within 1 week of the start of feeding and throughout the entire feeding period. However, when the daily intake of the combination of the heat‐killed lactic acid bacteria was 0.2 mg/kg of body weight per day, the values were elevated, though they were significantly lower than those of the control group. In other words, when the daily intake of the combination of the heat‐killed lactic acid bacteria was 0.05 and 0.1 mg/kg of body weight per day, a very high feeding attraction effect was observed. Furthermore, when the intake of bacteria was 0.050 or 0.1 mg, a higher feeding attraction effect was observed than when the intake of bacteria was 0.025 mg, and conversely, when the intake of bacteria was 0.2 mg, the feeding attraction effect was significantly lower than when the intake of bacteria was 0.05 or 0.1 mg.

**TABLE 5:** Effect of feed including different concentrations of a heat‐killed lactic acid bacteria mixture (the multispecies combination) with a ratio of *Lactobacillus delbrueckii* NBRC116386, *Leuconostoc mesenteroides* NBRC116387, and *Lactococcus lactis* NBRC116388 of 2:2:3 on the feeding attraction effect in coho salmon, *Oncorhynchus kisutch* (upper; the number of times each rank of the 28 surveys conducted, lower; statistical dataα).

Experimental group	Feeding completion rank				
	1	2	3	4	5
Control	0	1	4	12	11
25 M (multispecies combination of 0.025 mg/kg BW/day)	3	1	7	9	8
50 M (multispecies combination of 0.050 mg/kg BW/day)	9	14	3	2	0
100 M (multispecies combination of 0.10 mg/kg BW/day)	16	10	1	1	0
200 M (multispecies combination of 0.20 mg/kg BW/day)	1	2	13	3	9

	Control	25 M	50 M	100 M
25 M	0.25169	ー	ー	ー
50 M	2.74E‐08	3.63E‐05	ー	ー
100 M	3.48E‐09	1.65E‐06	0.5657535	ー
200 M	0.19418	0.99992	5.84E‐06	1.28E‐07

*Note*: The number before M indicates the amount of heat‐killed lactic acid bacterial powder (mg) to be administered to 1 kg of fish per day. The presence or absence of significant differences was determined using the Steel‐Dwass multiple comparison test (significance level α < 0.05).

**FIGURE 2:**
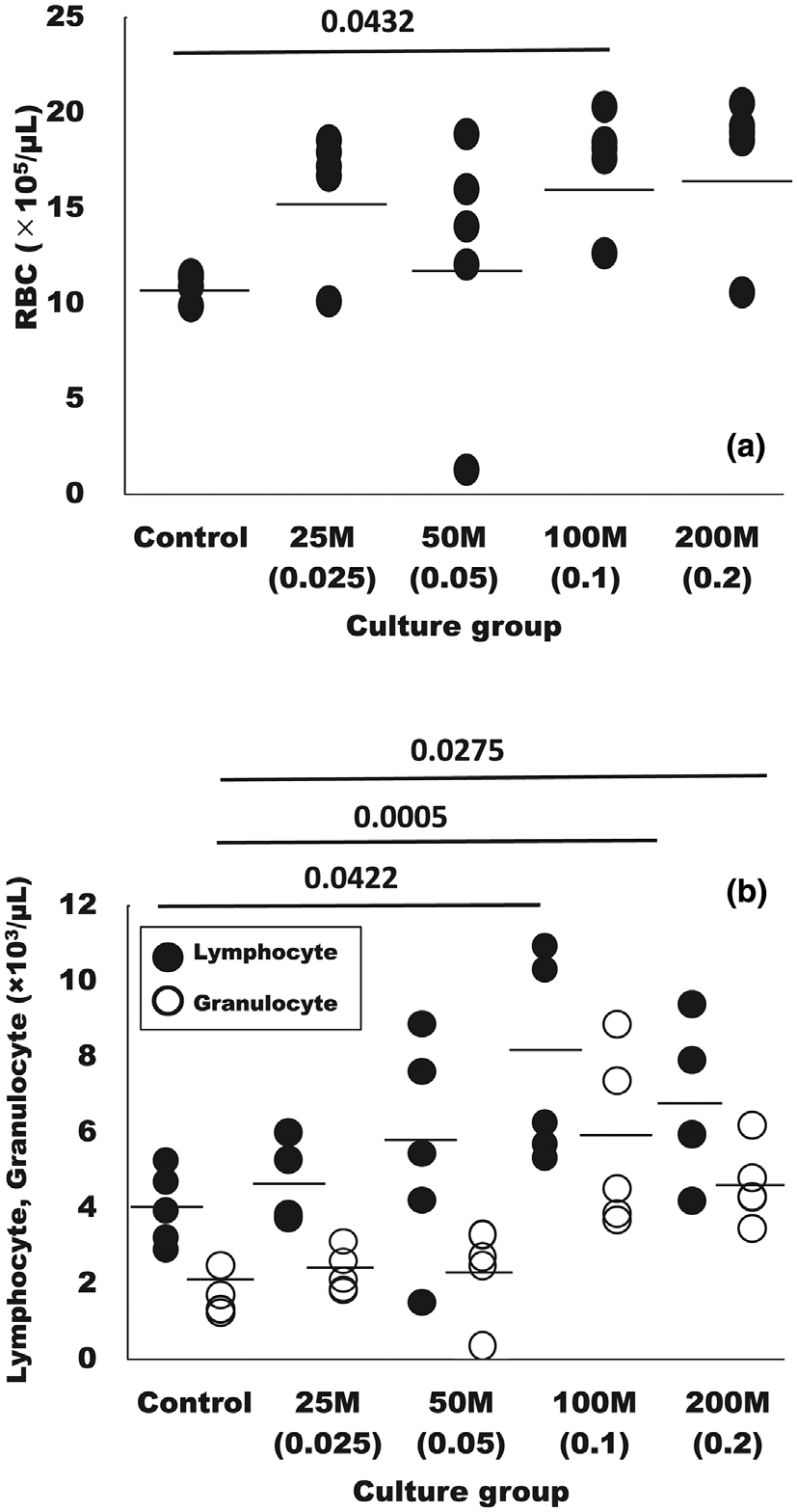
Effect of feed including the heat‐killed lactic acid bacteria mixture with a ratio of *Lactobacillus delbrueckii* NBRC116386, *Leuconostoc mesenteroides* NBRC116387 and *Lactococcus lactis* NBRC116388 of 2: 2: 3 on the number of red blood cells (a), granulocytes and lymphocytes (b) in coho salmon, *Oncorhynchus kisutch*. The number before M indicates the amount of heat‐killed lactic acid bacterial powder (mg) to be administered to 1 kg of fish per day. The number in parentheses indicates the amount of heat‐killed lactic acid bacteria (mg) to be administered to 1 kg of fish per day. Data are given as mean ± SD, *n* = 5. *p*‐value is by one‐way ANOVA with Tukey's post‐test for multiple comparisons.

Figure 2 shows the effect of feeding with different amounts of the combination of the heat‐killed lactic acid bacteria on the red blood cell (a), lymphocyte, and granulocyte counts (b) of coho salmon. The red blood cell count increased significantly when the daily intake of the combination was 0.1 mg/kg BW/day (*p* = 0.0432). The lymphocyte count was significantly higher at 0.1 mg/kg BW/day (*p* = 0.0422), compared to the control group. Granulocyte counts increased significantly at 0.1 mg and 0.2 mg/kg BW/day compared to the control group (*p* = 0.0005 and 0.0275, respectively). Also, significantly higher values were observed at 0.05 mg and 0.1 mg/kg BW/day compared to the 0.025 mg/kg BW/day group (*p* = 0.0018 and 0.0041, respectively).

**FIGURE 3:**
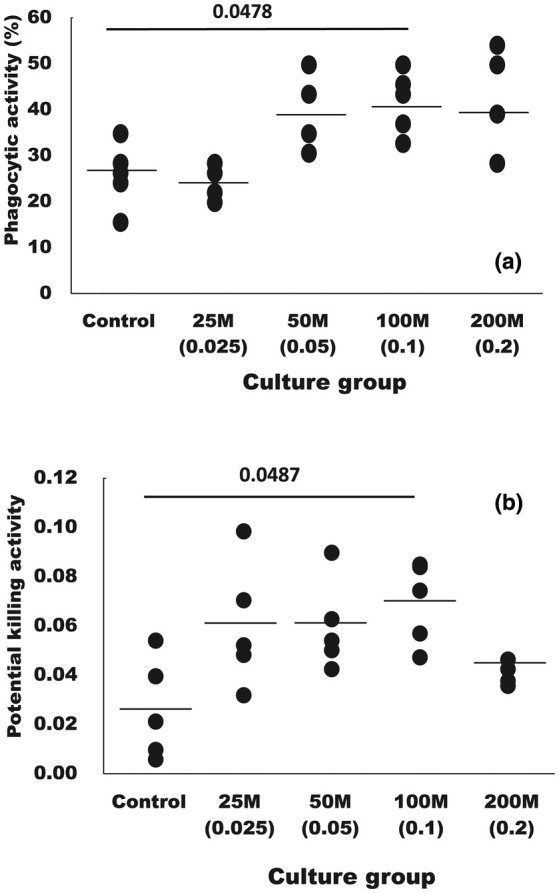
Effect of feed including the heat‐killed lactic acid bacteria mixture (multispecies combination) with a ratio of *Lactobacillus delbrueckii* NBRC116386, *Leuconostoc mesenteroides* NBRC116387, and *Lactococcus lactis* NBRC116388 of 2:2:3 on the phagocytic activity of granulocytes (a) and the potential killing activity (b) in coho salmon, *Oncorhynchus kisutch*. The number before M indicates the amount of heat‐killed lactic acid bacterial powder (mg) to be administered to 1 kg of fish per day. The number in parentheses indicates the amount of heat‐killed lactic acid bacteria (mg) to be administered to 1 kg of fish per day. Data are given as mean ± SD, *n* = 5. *p*‐ value is by one‐way ANOVA with Tukey's post‐test for multiple comparisons.

Figure 3 shows the effect of the administration of different amounts of the combination of heat‐killed lactic acid bacteria on the phagocytic activity of granulocyte (a) and the potential killing (PK) activity (b) in coho salmon. When the multispecies combination was administered at 0.1 mg/kg BW/day, both the granulocyte phagocytic activity and the PK activity were significantly increased compared to the control group (*p* = 0.0337). The values of the groups administered at 0.05, 0.1, and 0.2 mg/kg BW/day were also significantly higher than those of the group administered at 0.025 mg/kg BW per day (*p* = 0.0482, 0.0133 and 0.0281, respectively). The PK values were significantly higher when the daily intake of the combination was 0.025, 0.05, and 0.1 mg/kg body weight. The value was significantly higher when the combination was 0.1 mg/kg BW/day (*p* = 0.0487). When the daily intake of the combination was 0.025 mg/kg BW/day and 0.05 mg/kg BW/day, each average value was about three times higher than the control group, but no significant increase was observed.

Different doses of heat‐killed lactic acid bacteria (daily dose of heat‐killed lactic acid bacteria is 0, 0.025, 0.05, 0.1 and 0.2 mg/kg body weight, respectively) did not affect the skin lysozyme activity (⊿_620_/mg protein) of coho salmon. Data (*n* = 5) were of each group as follows: Control; 0.024 ± 0.021, 25 M; 0.059 ± 0.026, 50 M; 0.059 ± 0.019, 100 M; 0.069 ± 0.017, 200 M; 0.038 ± 0.005.

**FIGURE 4:**
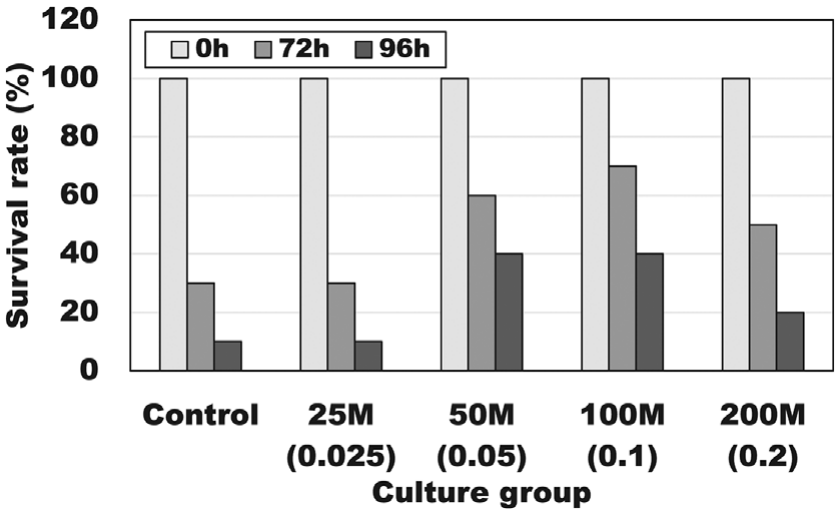
Effect of feed including the heat‐killed lactic acid bacteria mixture with a ratio of *Lactobacillus delbrueckii* NBRC116386, *Leuconostoc mesenteroides* NBRC116387, and *Lactococcus lactis* oki strain 3–1 of 2:2:3 on the survival rate in coho salmon, *Oncorhynchus kisutch*, held under a low dissolved oxygen condition (4.2 ± 0.2 mg/L) at 16°C. The number before M indicates the amount of heat‐killed lactic acid bacterial powder (mg) to be administered to 1 kg of fish per day. The number in parentheses indicates the amount of heat‐killed lactic acid bacteria (mg) to be administered to 1 kg of fish per day.

Figure 4 shows the effect of administration of different amounts of the combination of heat‐killed lactic acid bacteria on the survival rate of coho salmon exposed to a hypoxic condition. In a hypoxic (dissolved oxygen concentration of 4.2 ± 0.2 mg/L) challenge test, the survival rate after 3 days was higher (50%–70%) than the control group (30%) when the daily intake of the combination of heat‐killed lactic acid bacteria was 0.05, 0.1, and 0.2 mg/kg body weight for 1 kg body weight fish. Furthermore, the survival rate after 4 days from the start of hypoxic challenge was 40% when the daily intake of the combination of heat‐killed lactic acid bacteria was 0.05 and 0.1 mg/kg body weight, and 20% when the intake was 0.2 mg/kg body weight, both of which were higher than the 10% of the control group. Moreover, based on macroscopic observation, the amount of fluid in the digestive tracts of all fish, including dead fish, in the group administered heat‐killed bacteria was remarkably less than the same amount in all fish, including dead fish, in the control group.

## 4 DISCUSSION

It has been reported that administration of *Lactobacillus delbrueckii* subsp. *delbrueckii* (AS13B) via rotifers or *Artemia nauplii* modifies the intestinal flora of European sea bass (*Dicentrarchus labrax*, L.) larvae, leading to a decrease in cortisol content in the body and an increase in survival rate (Silvi et al., 2008). It has also been reported that feeding carp *Cyprinus carpio* with 1 × 10^6^ or 1 × 10^7^ CFU/g of *Lactobacillus delbrueckii* increases antioxidant enzyme activities such as superoxide dismutase (SOD), catalase (CAT), glutathione peroxidase (GPX), and total antioxidant capacity (T‐AOC), as well as increasing survival rates after challenge with *Aeromonas hydrophila* (Zhang et al., 2017). *Leuconostoc mesenteroides* has been reported to exhibit high inhibitory activity against food‐borne pathogens and spoilage microbial species (El‐Jeni et al., 2016). It has also been suggested that feeding with *Leuconostoc mesenteroides* subsp. *Jonggajibkimchii* DH was effective in restoring the innate immune system and resolving growth deficiency in juvenile loach (*Misgurnus anguillicaudatus*) by improving the abnormalities in the digestive tract caused by the ingestion of microplastics (Xia et al., 2024). In addition, it has been reported that ingestion of the exopolysaccharide (EPS) produced by *Leuconostoc mesenteroides* changes the host's gut microbial composition of intestinal bacteria and contributes to improving the host's glucose metabolism and energy homeostasis (Miyamoto et al., 2023). *Lactococcus lactis* is globally recognized as an effective microorganism for adjusting the intestinal microbial ecological balance of animals and improving the host immune function. For example, it has been reported that *Lactococcus lactis* intake (10^5^–10^7^ CFU/g feed) likely has beneficial effects on mandarin fish (*Siniperca chatsi*) growth, liver and intestinal health, *A. hydrophila* resistance, and gut microbial diversity, but these beneficial effects appear to be attenuated when the *Lactococcus lactis* level in the feed is increased to 10^7^ CFU/g and more (Zhu et al., 2021). This lactic acid bacterium is also known to produce nisin and suppress the growth of gram‐positive bacteria, including Listeria monocytogenes (Yamaki & Yamazaki, 2014). Therefore, it is expected that the intake of a multispecies combination of these bacteria will help regulate the physiological condition of fish and contribute to building bodies that are resistant to disease and stress.

To improve the health of fish using live lactic acid bacteria, however, it is necessary to feed the fish with the bacteria prepared according to the timing of feeding, which is very difficult in terms of cost and labor. It has been reported that it is difficult to establish live lactic acid bacteria in the intestines (Ohashi, 2006). However, the use of killed bacteria improves safety for the administered individual and the environment, and allows for a stable supply, which leads to improved convenience. killed bacteria is expected to have similar effects to those of live bacteria (Ditengou et al., 2023; Hassan et al., 2016; Izumo et al., 2011; Kakuta et al., 2023; Piqué et al., 2019; Vintiñi & Medina, 2011). Izumo et al. (2011) reported that heat‐killed *Lactobacillus pentosus* S‐PT84 strain is more effective than the live strain in inducing IL‐12 or IFN‐c production in vitro, and that it is important to use multiple strains selected for their immunomodulatory activity and adhesion properties to prepare the heat‐killed bacterial products. Other cases have been reported in which the administration of killed bacteria had significant benefits even when the administration of live bacteria did not produce the expected effect (Jin et al., 2020; Piqué et al., 2019).

When preparing killed bacteria, the concentration is often adjusted using dextrin or other substances. Since the bacteria sample used for this experiment had undergone the same processing process, the effect of administering dextrin was investigated in advance, but the administration of the dextrin used to prepare the heat‐killed lactic acid bacteria did not affect the feeding behavior or growth of the fish, or some of the physiological indicators. Therefore, it can be considered that the effect of administering the bacteria sample was brought about by the heat‐killed lactic acid bacteria.

When coho salmon were fed 0.1 mg/kg body weight/day of heat‐killed lactic acid bacteria, *Lactobacillus delbrueckii*, *Leuconostoc mesenteroides*, and *Lactococcus lactis*, either one of each or the multispecies combination (mixed at a 2:2:3 ratio) for 4 weeks, it was found that *Lactococcus lactis* increased the numbers of granulocytes, and that feeding the multispecies combination significantly increased the phagocytic activity of granulocytes (Experiment I).

In Experiment 2, 1 kg of coho salmon was fed the multispecies combination at 0, 0.025, 0.05, 0.1, and 0.2 mg/kg BW/day. As a result, it was found that a feeding attraction effect was observed at 0.05 mg/kg BW/day or more, and that an increase in red blood cell and lymphocyte counts was induced at 0.1 mg/kg BW/day, as well as a significant increase in phagocytic activity of granulocytes and PK activity. It was also found that most of the above‐mentioned effects, except the number of granulocytes, were reduced even if 1 kg of coho salmon was fed the combination at a dose of more than 0.2 mg per day.

At this time, it is unclear why *Lactococcus lactis* or the multispecies combination of the three types of lactic acid bacteria used in this study, especially the latter, were so effective, and the mechanism by which each killed bacteria exerts its effect. Regarding this, we think that it is possible that the high effectiveness of the study was achieved by the components contained in the cell walls of the three types of lactic acid bacteria, such as peptidoglycan, lipoteichoic acid, and nisin (in the case of *Lactococcus lactis*), which act in coordination with immune components such as IgT in the intestine to activate immune responses, suppressing the growth of bad bacteria in the intestine and improving the intestinal environment, while the heat‐sterilized bacteria themselves adhere to the intestinal wall to form a temporary barrier, preventing bad bacteria from adhering to the intestinal wall and entering the body. However, since the presence of Peyer's patches has not been confirmed in the intestines of fish, it is possible that an immune response mechanism activated by the administration of killed lactic acid bacteria, different from that in mammals, is at work. In the future, it will be necessary to investigate why feed containing the heat‐killed lactic acid bacteria stimulates the feeding behavior of fish, as well as the effects of administering the combination of these bacteria on the intestinal bacterial flora and the amount and quality of mucus, antibody appearance status, the quality and quantity of fermented products such as organic acids and fatty acids produced in the intestinal tract, and the transport of these substances through the intestinal wall.

In addition, the increase in survival rate during exposure to hypoxia revealed by this test (Figure 4), that is, the provision of hypoxia tolerance, could be a significant countermeasure against the decline in physiological function and feeding behavior of farmed fish caused by the rise in seawater temperature and the decrease in oxygen concentration in water due to global warming. Although the reason for the enhancement of hypoxia tolerance by administration of the heat‐killed bacteria combination is unclear, it is thought to have been achieved by the combined action of the increase in red blood cell count (Figure 2a), the increase in oxygen intake efficiency in the gills, the calming under hypoxia, the suppression of excessive stress response and swimming behavior, and so on. It is possible that the suppression of excessive stress response, that is, the optimization of stress response, was also related to the improvement of the survival rate of the group that received the same bacteria under hypoxic conditions. Stress increases plasma cortisol concentrations in fish. Cortisol administration significantly increased oxygen consumption and plasma glucose levels in FW trout (Morgan & Iwama, 1996). In other words, stressed fish with high plasma cortisol levels are known to consume more oxygen than healthy fish (Barton & Schreck, 1987; Randall & Perry, 1992). Freshwater fish do not normally drink water, but are known to drink water when they are held under stress conditions (Huising et al., 2003; Nobata et al., 2013; Takei & Hirose, 2002). Further detailed investigation is needed into changes in the catecholamine and cortisol concentrations in the blood of fish, as well as changes in drinking and swimming behavior during exposure to hypoxia. However, the results of this experiment, in which the amount of freshwater stored in the digestive tract of fish in the group administered heat‐killed bacteria combination was remarkably smaller than that in the control group, support the conclusion that the intake of the killed lactic acid bacteria complex suppressed the excessive stress response of fish.

The results of Experiments I and II above show that the administration of a multispecies combination of at least three types of heat‐killed lactic acid bacteria, *Lactobacillus delbrueckii* NBRC116386, *Leuconostoc mesenteroides* NBRC116387, and *Lactococcus lactis* NBRC116388, can exert a wide range of effects at a lower dose than the administration of the three types of heat‐killed lactic acid bacteria alone. In addition, in a 4‐week feeding test, the dose of the heat‐killed bacteria required to elicit the effect was 0.05–0.1 mg/kg body weight/day; this amount corresponds to 2.5–5.0 × 10^9^ bacteria (the amount of heat‐killed bacteria added to the feed when the daily feeding amount was 2% of body weight was 2.5–5.0 mg/kg feed), which was smaller than the doses of killed bacteria previously reported for animals including fish. An example for comparison is described below. Oral administration of 2 or 17 mg/kg body weight of heat‐killed *Enterococcus faecalis* EF‐2001 to mice with colitis induced by rectal administration of dinitrobenzene sulfonic acid (DNBS) reduced the expression of several cytokines, including cyclooxygenase (COX)‐2, inducible nitric oxide synthase (iNOS), interferon (IFN)‐γ, interleukin (IL)‐1β, and IL‐6, in the inflamed colon and inhibited colonic tissue destruction compared to the DNBS‐only treatment group (Choi et al., 2019). When healthy individuals ingest 10 mg of heat‐killed *Lactobacillus plantarum* L‐137 bacteria daily for 12 weeks, that is, 0.2 mg/kg body weight, it promotes T cell proliferation along with an increase in short‐chain fatty acids and *Bacteroidetes* in the intestinal tract (Nakai et al., 2021). Hien et al. (2021) reported that the administration of feed containing 10 mg/kg of heat‐killed *Lactobacillus plantarum* L‐137 effectively improved the growth and immunity of juvenile giant catfish (*Clarias macrocephalus*). Wang et al. (2022) fed food containing *Pseudoalteromonas piscicida* 2515 (live and heat‐killed) to juvenile flounder (*Paralichthys olivaceus*) and checked its safety as well as examined changes in immune response and intestinal flora. They showed that the administration of feed containing live bacteria at 10^10^/kg of feed or heat‐killed bacteria at 10^8^ CFU/kg of feed both enhanced nonspecific immunity and improved the intestinal function of fish, though the administration of live bacteria at 10^12^ CFU/kg of feed may cause hemolytic activity. When tilapia was fed feed containing heat‐killed *Lactobacillus plantarum* L‐137 bacteria three times a day under satiation conditions for 90 days, 10–20 mg/kg feed was effective in supporting growth, and 250 mg/kg feed was recommended to prevent *Streptococcus agalactiae* infection (Ekasari et al., 2023).

It is believed that the administration of preventive and therapeutic vaccines is effective for the prevention and treatment of specific pathogens, respectively (Delany et al., 2014; Li et al., 2023). However, there are few approved commercially available vaccines, and the vaccines that can be used are limited in the types of diseases. Moreover, the breeding conditions of the target fish species, and depending on the usage method, they may not necessarily be highly effective (Wakabayashi, 2002). Therefore, we believe that it is extremely significant to administer appropriate types and amounts of killed lactic acid bacteria or mixtures of the same, as presented in this study, to fish species for aquaculture, in order to maintain their health and increase their nonspecific biological defense activity, as well as to reduce side effects of vaccines administered when specific infectious diseases spread or when signs of infection are observed.
